# Anatomical description of the ventral and dorsal cervical rootlets in rats: A microsurgical study

**DOI:** 10.1590/acb370307

**Published:** 2022-06-01

**Authors:** Deivid Ramos dos Santos, Nayara Pontes de Araújo, Renan Kleber Costa Teixeira, Lívia Guerreiro de Barros Bentes, Dante Bernardes Giubilei, Rosa Helena de Figueiredo Chaves, Arnaldo Algaranhar Gonçalves, Edson Yuzur Yasojima, Rui Sergio Monteiro de Barros

**Affiliations:** 1 MD. Universidade do Estado do Pará – Postgraduate Program in Surgery and Experimental Research – Belém (PA), Brazil.; 2 Graduate student. Universidade do Estado do Pará – School of Medicine – Department of Experimental Surgery – Belém (PA), Brazil.; 3 MD. Universidade do Estado do Pará – School of Medicine – Department of Spine Surgery – Belém (PA), Brazil.; 4 PhD. Universidade do Estado do Pará – Faculty of Veterinary Medicine – Department of Experimental Surgery – Belém (PA), Brazil.; 5 PhD. Associate Professor. Universidade do Estado do Pará – School of Medicine – Department of Experimental Surgery – Belém (PA), Brazil.

**Keywords:** Spinal Cord, Nervous System, Spinal Nerve Roots, Cervical Cord, Rats

## Abstract

**Purpose:**

To describe the anatomical aspects of the cervical rootlets and to quantify the number of rootlets that compose C1 to T1.

**Methods:**

Twenty male rats were used in this study. The dorsal rootlets from C1 to T1 were analyzed. To study the ventral rootlets, the posterior root avulsion was performed using a microhook, allowing exposure of the ventral roots through manipulation of the denticulate ligament and arachnoid mater. The parameters analyzed were the number of ventral and dorsal rootlets by side and level.

**Results:**

The formation of the respective spinal nerve was observed in the spinal roots the union of the ventral and dorsal roots. In four animals the C1 spinal root had no dorsal and/or ventral contribution. There is no normal pattern of numerical normality of the dorsal and ventral rootlets. The average number of fascicles per root was 4.08, with a slight superiority on the left side. There was a slight superiority of the dorsal rootlets compared to the ventral rootlets.

**Conclusions:**

This investigation was the first to study cervical rootlets in rats. In 20% of the sample studied, the dorsal root of C1 was absent mainly on the left side. There is a nonlinear numerical increase from C1 to T1 in the rootlets. There is a numerical predominance of cervical fascicles on the left side, confronting several studies related to the functional predominance of right laterality, requiring new studies that correlate these variables.

## Introduction

Traumatic spinal cord injuries have gained prominence in recent years, especially among automobile victims, because of the severe neurological sequelae resulting from these accidents^1^. It is more frequent among young people and has a significant economic and social impact, requiring precise clinical and surgical management^2^. It is estimated that 100,000 patients per year require nerve repair surgery in the United States and Europe, making it a significant public health problem^3^.

The surgical management of these patients requires detailed knowledge of medical anatomy and physiology, creating the need to develop new surgical techniques for repair and evaluation after nerve injury that can be obtained from clinical trials and experimental work^4^^,^ 5.

In the field of experimentation, the rat stands out as an important model used especially in microsurgery, due to its easy acquisition and handling, it does not require large physical spaces, and the anesthetic protocols and the physiological parameters of the species are well known, allowing for a decrease in secondary surgical complications^6^. And, mainly, this model has great anatomical similarities with the human, allowing several comparisons^7^^,^ 8.

This similarity allows the use of rat in a wide variety of experimental fields when trying to solve phenomena relevant to human medicine such as the recovery, even partially, of functional abilities lost after nerve injury^9^^,^ 10.

Despite the great importance of the rat as a model for animal experimentation, no studies were found that addressed microsurgical anatomical details on the rootlets (radicular fascicles) of the rat spinal cord^8^^,^ 9.

Knowing the large gap in information about the detailed anatomy of the origin of the rootlets that make up the cervical spinal nerves, the objective of this work was to describe the microsurgical anatomic aspects of the ventral and dorsal cervical rootlets in rats. This shows a still little explored field and scarce information on this theme that can subsidize recent research on neural reimplantation treatments at the spinal cord level.

## Methods

The study followed the rules set out in the Brazilian legislation on animal care (Law No. 11,794/08), which is based on NIH guidelines, and complied with the Council for International Organization of Medical Sciences ethical code for animal experimentation and the ARRIVE guidelines. The project was approved in advance by the Animal Use and Care Committee of the Universidade do Estado do Pará (Protocol No. 24/18).

Twenty male Wistar rats (12–15 weeks old), with no veterinary diseases, weighing 220–280 g, were used in this study, already euthanized from other experimental studies, without previous surgical interventions on the central and peripheral nervous system, respecting the principle of reduction^10^.

### Experimental procedure

All animals were submitted to the same protocol. Initially, the cervical region was shaved. The animals were placed in a horizontal ventral position above plastic support on the cervical region to flex the spine to increase the interlaminar space. Then, a single, vertical access route was carried out between the meeting of the occipital bone and the nuchal line to the region of the second thoracic vertebra.

**Figure 1:**
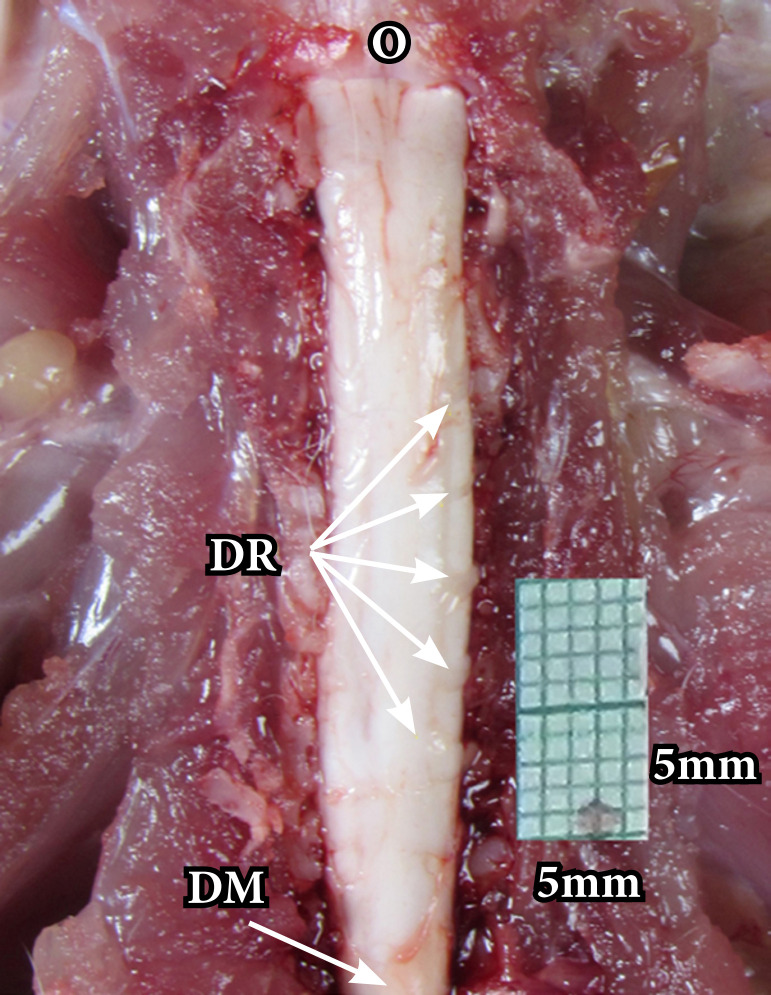
Posterior (dorsal) access route of the spinal cord of rats with exposure of the posterior roots.

The subcutaneous tissue was carefully laid back, and there was a blunt dissection of the paraspinal muscles to expose the cervical spine. The spinous process of T2 was used as an anatomical landmark to identify the others vertebras. The paraspinal muscles were pushed laterally with retractors until the complete visualization of the spinous processes of the spinal vertebrae. For access to the spinal cord, a Kerrison instrument was used for bilateral transection of the C1 to T1 vertebral arches (Fig. 1).

O: occipital region; DR: dorsal rootlets; DM: dura mater.

The procedures were performed under a video system composed by a high-definition Sony camcorder HDR-CX 150 set to 62× magnification, connected with a 4K 55-inch curved television, positioned in front of the surgeon, by a digital HDMI cable^11^. Two white led light sources were used near the camera to provide adequate illumination of the operative field.

**Figure 2:**
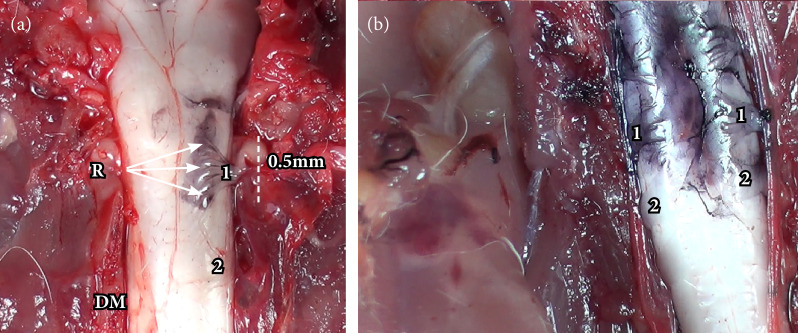
**(a)** Posterior (dorsal) radicular fascicles painted with India ink. **(b)** 1: fascicles stained in C5; 2: fascicles without stain in C6 demonstrating the difficulty in counting fascicles.

There was a blunt dissection between the dura mater and the arachnoid mater to expose the medulla and the roots. To count the fascicles, India ink was carefully applied on the surface of each fascicle with a microswab, as it is possible to observe the difference between stained (Fig. 2a) and nonstained fascicles (Fig. 2b). So, the dorsal rootlets from C1 to T1 on both sides were analyzed.

R: rootlets after staining in C1; DM: dura mater.

The posterior root avulsion using a microhook was performed to study the ventral rootlets, allowing to exposure the ventral roots through manipulation of the denticulate ligament and arachnoid mater. The parameters analyzed were the number of ventral and dorsal rootlets by side and level (C1 to T1 vertebra). BioEstat 5.4 software was used for analyses. The Student’s t-test was used to compare the numeric variables.

Each surgical procedure was performed in one day by the same surgeon, in the surgery center during the morning shift, supported by a veterinarian. The roots’ counting was performed by two researchers, and in case of divergence, a third researcher revised it.

## Results

### External medullar morphology

**Figure 3:**
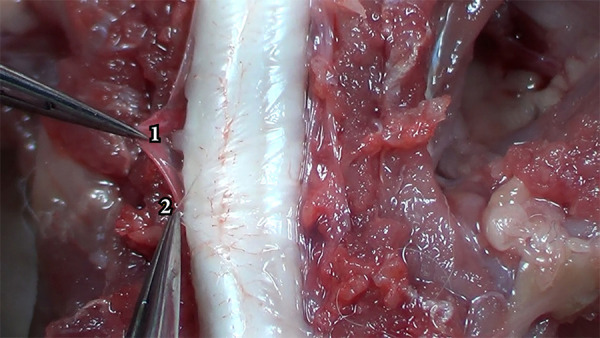
Differentiation of the dura mater (1) from the arachnoid mater (2).

The Wistar’s spinal cord is flat and cylindrical, protected by vertebrae, muscles, ligaments, meninges, and cerebrospinal fluid. After the vertebrectomy, it’s possible to observe the dura mater with elastic consistency, resistance, thick and slightly transparent, followed by the arachnoid space and the arachnoid mater, friable, thin, and not very resistant to microsurgical manipulation (Fig. 3). It was not possible to observe the pia mater in the videomagnification system adopted^12^^,^ 13.

The meninges are made up of three layers: the dura mater (outermost layer), the arachnoid membrane, and the pia mater (innermost layer). The junction of the arachnoid and pia mater is called the leptomeninges, which are held together by arachnoid trabeculae. This arrangement forms a compartment called the subarachnoid space where the cerebrospinal fluid flows and which also contains blood vessels and nerves^13^^,^ 14.

The dura mater is thicker and consists of a thicker collagenous membrane that is more resistant to microsurgical manipulation. The surfaces of the dura mater and arachnoid slide over each other and it is possible to microsurgically remove the dura mater without damaging the adjacent meningeal. The pia mater is strongly adhered to the spinal cord and has a large number of blood vessels that nourish the underlying nerve tissue (Fig. 3)^12^^–^ 15.

### Cervical rootlets

In the spinal roots, the union of the ventral and dorsal roots was observed for the formation of the respective spinal nerve. In four animals the C1 spinal root had no dorsal contribution, mainly on the left side.

**Figure 4:**
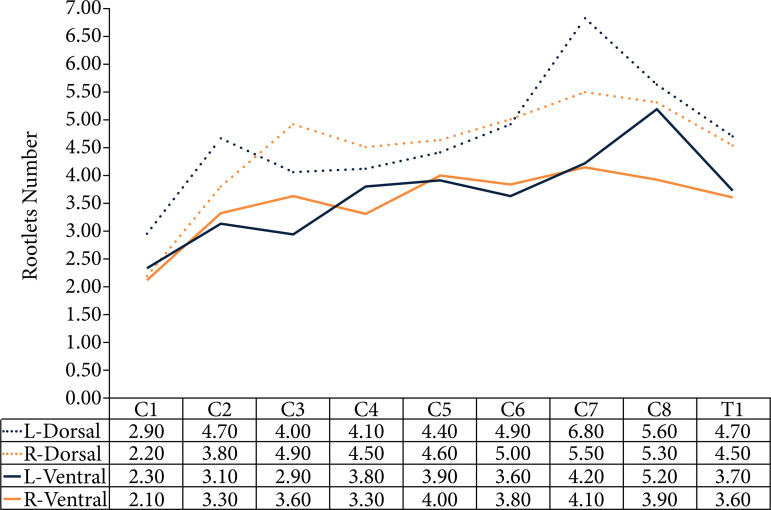
Numerical distribution of rootlets filaments by level and laterality. R: right; L: left.

It was observed that there is no normal pattern of numerical normality of radicular fascicles of the posterior and anterior roots (Table 1). The average number of fascicles per root was 4.08, with a slight superiority on the left side about the right. There was a superiority of the dorsal fascicles (4.58) compared to the ventral (3.58) (Table 2). There was no statistical difference between the variables studied in this research (p > 0.05). When separating by region, a numerical increase was identified from C1 to C8 in both anterior and posterior fascicles (Fig. 4).

**Table 1:** Numerical distribution of rootlets per rat by laterality.

Vertebral levels	Average			
		Total	R	L
C1	D	2.55	2.20 ± 2.20	2.90 ± 1.83
	V	2.20	2.10 ± 1.10	2.30 ± 1.22
C2	D	4.25	3.80 ± 1.35	4.70 ± 1.08
	V	3.20	3.30 ± 0.84	3.10 ± 1.27
C3	D	4.45	4.90 ± 1.34	4.00 ± 1.81
	V	3.25	3.60 ± 0.83	2.90 ± 1.50
C4	D	4.30	4.50 ± 0.94	4.10 ± 1.63
	V	3.55	3.30 ± 1.33	3.80 ± 1.55
C5	D	4.50	4.60 ± 1.74	4.40 ± 1.69
	V	3.95	4.00 ± 0.94	3.90 ± 1.41
C6	D	4.95	5.00 ± 1.45	4.90 ± 1.73
	V	3.70	3.80 ± 1.36	3.60 ± 1.78
C7	D	6.15	5.50 ± 1.99	6.80 ± 2.20
	V	4.15	4.10 ± 1.25	4.20 ± 1.76
C8	D	5.45	5.30 ± 1.85	5.60 ± 2.00
	V	4.55	3.90 ± 1.99	5.20 ± 2.17
T1	D	4.60	4.50 ± 2.00	4.70 ± 1.80
	V	3.65	3.60 ± 2.00	3.70 ± 2.15

C: cervical spinal rootlets; T: thoracic spinal root; R: right; L: left; D: dorsal; V: ventral.

**Table 2:** Distribution of mean rootlets fascicles by laterality.

Division	Right	Left	Average
Ventral	3.52	3.63	3.58
Dorsal	4.48	4.68	4.58
Average	4.00	4.15	4.08

## Discussion

Different species of animals are used in the study of nerves, including primates, dogs, cats, rats, mice, and rabbits^1^^–^ 3 ^,^ 16. The choice of the rat was due to ease of access and handling^17^. These animals have anatomical structures similar to humans and suitable for various studies^5^^–^ 8. It is worth mentioning that there are no published studies on the counting of sensory and motor fascicles in rats since the few existing studies were performed in large animals, and data on the most frequently used animal, the rat.

The current study aimed to investigate the anatomy of the existing neural structures from C1 to T1. It is emphasized that this is the first study to quantify the radicular fascicles that forms the ventral and dorsal roots in rats. The videomagnification system used has been shown to offer reliable images with high contrast resolution and improved accuracy to quantify neural anatomy^11^^,^ 18.

More importantly, these results aim to improve the understanding of the anatomical variations along the cervical spine when considering a vertebrectomy or laminectomy procedure, as the major difficulty in performing these procedures by posterior approach is the preservation of delicate neural structures during surgical dissection. Such a methodological approach has been unexplored so far and will serve as support for several experimental studies that aim to achieve surgical repair and functional recovery of the affected nerve.

### Origin of cervical spinal roots

The cervical nerves arise from the spinal cord in the form of rootlets, or fila radicularia or radicular fascicles, smaller neuron bundles that coalesce to form roots. For each spinal nerve, a ventral and dorsal root join to form the completed nerve^19^.

The origin of the spinal nerves reveals the segmentation of the spinal cord. They are formed by the union of the dorsal root, which is composed almost exclusively of afferent fibers (dorsal or sensory branch), and the ventral root, which is composed of efferent fibers (ventral or motor branch). Thus, the spinal nerves are arranged in pairs^12^^–^ 15.

Each spinal nerve leaves the spinal canal through the appropriate intervertebral foramen^13^. Thus, each cervical spinal nerve emerges cranially to the vertebra with the same designation^14^^,^ 15. It is observed that the cervical roots are short and have an approximately horizontal path toward the lateral region of the vertebral canal and its corresponding foramen, very similar to that found in several mammals and humans^12^^,^ 13.

It is important to note that the C1 spinal root had no dorsal contribution in four animals, and there was no ventral contribution from the same spinal level in one animal. This finding is similar to that found in the literature, especially in cadaveric studies developed in humans, where there is a rate of up to 28% of the absence of sensory contribution from the C1 roots^12^. There is no unanimity to state whether there is a contribution of the posterior root in the formation of the C1 spinal nerve. Therefore, larger studies are needed to establish a standard of normality in the rat.

### Radicular fascicles (rootlets)

No pattern of numerical normality was found in the fascicles of the spinal roots studied. There is scarce scientific data to compare the results found in this study, mainly related to the quantification of rootlets that originate the spinal nerves. This is justified by the great difficulty in performing the access route and counting structures smaller than 0.5 mm in width without damaging the neural tissue. In addition, the neural tissue reflects the external light from the videomagnification model preventing proper counting of rootlets.

Strategically, it was decided to dye the fascicles with India ink. It adheres to the surface and concentrates more in the spaces between the fascicles, delimiting and allowing the correct counting of these structures^20^. Despite making this study possible, it is important to develop further research to verify which is the most appropriate coloration and type of ink to help count the fascicles.

The number of rootlets was more numerous in C7 (6.8 ± 2.2) and C8 (5.6 ± 2.0), both in the left dorsal root. Because this study was the first performed in rats, it was not possible to compare the results with rats or small animals, so they were compared with studies performed in human cadavers^21^^–^ 23.

The results obtained in this study agreed with the study by Aydoğmuş and Çavdar^21^, and Karatas *et al*.^22^ who reported that the maximum number of sensory rootlets occurred at C6, C7, and C8. Liu *et al*.^23^ counted the number of rootlets in the motor and sensory roots by histological sections and reported that the dorsal root C7 contained the highest number of nerve fibers among the cervical roots, disagreeing with the results found in this work, given that the highest number found in the motor fascicles was in C8 (5.2 ± 2.17).

The result of the present study was consistent with Aydoğmuş and Çavdar^21^, and Karatas *et al*.^22^ but contradicts Liu *et al*.^23^. The difference may be related to the different object of study, which in this case is the rat, and by the different methods used for data collection, since the access route in our study was only through the posterior approach and the counting of motor fascicles was performed after the section of sensory fascicles.

The increased number of fascicles that make up the spinal nerves of the cervical and brachial plexus may be explained by the need for a large number of neural tissues to supply the forelimbs of the animals^17^^,^ 24 ^,^ 25.

Promising studies show that root reimplantation techniques may become an option in the treatment of brachial plexus injuries in the not-too-distant future^24^^,^ 25. Some experimental research in laboratory animals indicates that reimplantation of only 40% of the roots supplies the minimum requirement for functional recovery after nerve tissue injury^5^^,^ 26 ^,^ 27.

The results showed a greater number of root fascicles on the left side compared to the right side. Despite the result found, it was not possible to establish a correlation with the predominance of functional laterality in the studied animals because no functional test was performed before surgical dissection^17^. However, it is known that some studies show the existence of right-handed predominance in rats, so it would be expected a greater quantitative number of fascicles on the right side and not on the left^28^^,^ 29. Thus, further studies are needed to establish whether there is a correlation between the number of fascicles and the predominance of laterality in rats.

The study helps to understand the microanatomy of the root fascicles of the rat cervical spinal cord, subsidizing research on functional recovery of nerves implanted directly into the spinal cord after preganglionic neural injury, subsidizing new treatments such as nerve transfer in the spinal cord.

## Conclusions

This investigation was the first to study the root fascicles that form the cervical spinal nerves in rats. In 40% of the sample studied, the dorsal root of C1 is absent mainly on the left side. There is a nonlinear numerical increase from C1 to C8 in the rootlets. There is a numerical predominance of cervical fascicles on the left side.
